# Binimetinib in combination with nivolumab or nivolumab and ipilimumab in patients with previously treated microsatellite-stable metastatic colorectal cancer with *RAS* mutations in an open-label phase 1b/2 study

**DOI:** 10.1186/s12885-024-12153-5

**Published:** 2024-04-11

**Authors:** Elena Elez, Antonio Cubillo, Pilar Garcia Alfonso, Mark R. Middleton, Ian Chau, Baha Alkuzweny, Ann Alcasid, Xiaosong Zhang, Eric Van Cutsem

**Affiliations:** 1 Medical Oncology Department, Vall d’Hebron University Hospital and Vall d’Hebron Institute of Oncology, Universitat Autònoma de Barcelona, Barcelona, Spain; 2 Centro Integral, Oncológico Clara Campal, HM CIOCC, Madrid, Spain; 3 Facultad HM Hospitales de Ciencias de La Salud UCJC, 28050 Madrid, Spain; 4 Medical Oncology Service, Hospital General Universitario Gregorio Marañón, Instituto de Investigación Sanitaria Gregorio Marañón (IiSGM), Universidad Complutense, Madrid, Spain; 5 Department of Oncology, NIHR Biomedical Research Centre, University of Oxford, Oxford, UK; 6 Gastrointestinal Unit, Royal Marsden Hospital, London & Surrey, UK; 7 Formerly Pfizer, Inc, San Diego, CA USA; 8 Pfizer Inc, Collegeville, PA USA; 9 Pfizer, Inc, New York, NY USA; 10 University Hospitals Gasthuisberg Leuven and KU Leuven, Leuven, Belgium

**Keywords:** Colorectal cancer, MSS, RAS, MEK1, Binimetinib, Nivolumab, Ipilimumab

## Abstract

**Background:**

In patients with previously treated *RAS*-mutated microsatellite-stable (MSS) metastatic colorectal cancer (mCRC), a multicenter open-label phase 1b/2 trial was conducted to define the safety and efficacy of the MEK1/MEK2 inhibitor binimetinib in combination with the immune checkpoint inhibitor (ICI) nivolumab (anti–PD-1) or nivolumab and another ICI, ipilimumab (anti-CTLA4).

**Methods:**

In phase 1b, participants were randomly assigned to Arm 1A (binimetinib 45 mg twice daily [BID] plus nivolumab 480 mg once every 4 weeks [Q4W]) or Arm 1B (binimetinib 45 mg BID plus nivolumab 480 mg Q4W and ipilimumab 1 mg/kg once every 8 weeks [Q8W]) to determine the maximum tolerable dose (MTD) and recommended phase 2 dose (RP2D) of binimetinib. The MTD/RP2D was defined as the highest dosage combination that did not cause medically unacceptable dose-limiting toxicities in more than 35% of treated participants in Cycle 1. During phase 2, participants were randomly assigned to Arm 2A (binimetinib MTD/RP2D plus nivolumab) or Arm 2B (binimetinib MTD/RP2D plus nivolumab and ipilimumab) to assess the safety and clinical activity of these combinations.

**Results:**

In phase 1b, 21 participants were randomized to Arm 1A or Arm 1B; during phase 2, 54 participants were randomized to Arm 2A or Arm 2B. The binimetinib MTD/RP2D was determined to be 45 mg BID. In phase 2, no participants receiving binimetinib plus nivolumab achieved a response. Of the 27 participants receiving binimetinib, nivolumab, and ipilimumab, the overall response rate was 7.4% (90% CI: 1.3, 21.5). Out of 75 participants overall, 74 (98.7%) reported treatment-related adverse events (AEs), of whom 17 (22.7%) reported treatment-related serious AEs.

**Conclusions:**

The RP2D binimetinib regimen had a safety profile similar to previous binimetinib studies or nivolumab and ipilimumab combination studies. There was a lack of clinical benefit with either drug combination. Therefore, these data do not support further development of binimetinib in combination with nivolumab or nivolumab and ipilimumab in *RAS*-mutated MSS mCRC.

**Trial registration:**

NCT03271047 (09/01/2017).

**Supplementary Information:**

The online version contains supplementary material available at 10.1186/s12885-024-12153-5.

## Background

Colorectal cancer (CRC) is the third most diagnosed cancer (10% of cancer cases) and the second leading cause of cancer-related deaths (9.4% of cancer deaths) worldwide [1]. Patients with early-stage CRC can usually be cured through surgical resection of the primary tumor, but patients with metastatic CRC (mCRC) have a 5-year survival rate of only 14% [2, 3]. CRC is a highly heterogeneous disease with different tumor phenotypes, each with specific molecular and morphological characteristics [4]. Due to these specific characteristics, CRC can be divided into discrete subclasses based on integrated molecular and clinical studies.

Comprehensive sequencing and proteomic studies have helped to define molecular subclasses of CRC, including disease that is microsatellite stable (MSS) or microsatellite-instability-high (MSI-H) [4–6]. Data has shown that MSS mCRC tumors have a distinct etiology and treatment recommendations that differ from tumors classified as MSI-H mCRC [5–7]. In general, immune checkpoint inhibitors (ICIs) are effective for MSI-H but not for MSS mCRC tumors [8]. However, approximately 96% of patients with mCRC have tumors with an ICI monotherapy–resistant MSS phenotype [7, 8]. Multifactorial mechanisms may contribute to the intrinsic resistance of MSS mCRC tumors to ICI therapy such as having a lower tumor mutational burden and being poorly immunogenic [9]. Furthermore, MSS mCRC tumors may have an immunosuppressive environment caused by increased levels of tumor-associated macrophages and regulatory T cells compared with MSI-H mCRC tumors [10].

In MSS mCRC, *RAS* mutations have been linked to more aggressive tumor biology and a shorter overall survival (OS) compared with *RAS* wild-type MSS mCRC [11, 12]. RAS belongs to a family of small G proteins, including KRAS, NRAS, and HRAS, that is responsible for controlling signaling downstream of ligand-dependent receptor activation. In mCRC overall, *KRAS* and *NRAS* activating mutations are reported in 40% and 3% to 5% of cases, respectively; *HRAS* activating mutations have been reported in rare cases [7, 13]. *RAS* mutations represent a clinical setting where MAPK pathway inhibition may positively modulate the efficacy of ICIs in patients with MSS mCRC. Several preclinical studies suggest that MAPK signaling may influence tumor immune escape mechanisms, including downregulation of major histocompatibility complex class 1 expression and upregulation of immunosuppressive cytokines and cell surface molecules, including PD-1 expression, which can increase T-cell infiltration into tumors and enhance the antitumor activity of PD-1 inhibitors [14, 15]. Therefore, combining a MEK1/MEK2 inhibitor with ICI treatment might be a way to overcome the inherent resistance of MSS mCRC to ICI.

Binimetinib (also known as MEK162 or ARRY-438162) is a potent and selective allosteric, ATP-uncompetitive inhibitor of MEK1 and MEK2 [16]. In most cancers, the ERK pathway, including RAS, BRAF, CRAF, and MEK1 or MEK2, is hyperactive due to deregulation of receptor tyrosine kinases. MEK1 and MEK2 are uniquely positioned within the ERK pathway, where they process inputs from multiple upstream activating kinases following RAF activation, making them attractive drug targets. Binimetinib has been investigated both as a single agent and in combination with other agents in patients with selected advanced or metastatic CRC [16, 17]. The first in-human trial of binimetinib identified 60 mg twice daily (BID) as the maximum tolerated dose (MTD) and 45 mg BID as the recommended phase 2 dose (RP2D) for binimetinib monotherapy in patients with BRAF-mutant CRC [16]. Preliminary results indicate that binimetinib treatment in combination with ICIs has encouraging activity and acceptable tolerability in patients with MSS mCRC [18, 19].

Nivolumab is an ICI monoclonal antibody (mAb) that targets the PD-1 cell surface membrane receptor. Nivolumab monotherapy and nivolumab in combination with the ICI ipilimumab, a mAb that targets CTLA4, have been approved to treat patients with MSI-H/mismatch repair-deficient (dMMR) mCRC that has progressed following treatment with fluoropyrimidine, oxaliplatin, and irinotecan [20, 21]. MSS mCRC tumors generally have fewer infiltrative CD8+ T-cell populations than MSI-H tumors [22], and immuno-oncological approaches with checkpoint inhibition alone may be insufficient for patients with limited tumor immune cell infiltration. The combination of MEK inhibitors with PD-1 inhibitors and CTLA-4 inhibitors has synergically promoted durable tumor regression and longevity of tumor-infiltrating CD8+ T cells to provide additional efficacy in preclinical mouse models [14, 15].

Given that MAPK pathway inhibition might overcome the resistance to ICI in patients with MSS mCRC harboring an activating *RAS* mutation, this multicenter, open-label, phase 1b and phase 2 trial was designed to determine the MTD, RP2D, and schedule of binimetinib treatment in combination with nivolumab with or without ipilimumab. This study was also designed to assess the safety and efficacy of binimetinib administered in combination with nivolumab or nivolumab and ipilimumab in patients with previously treated MSS mCRC with a documented *RAS* mutation.

## Methods

### Study design and participants

**Fig. 1:**
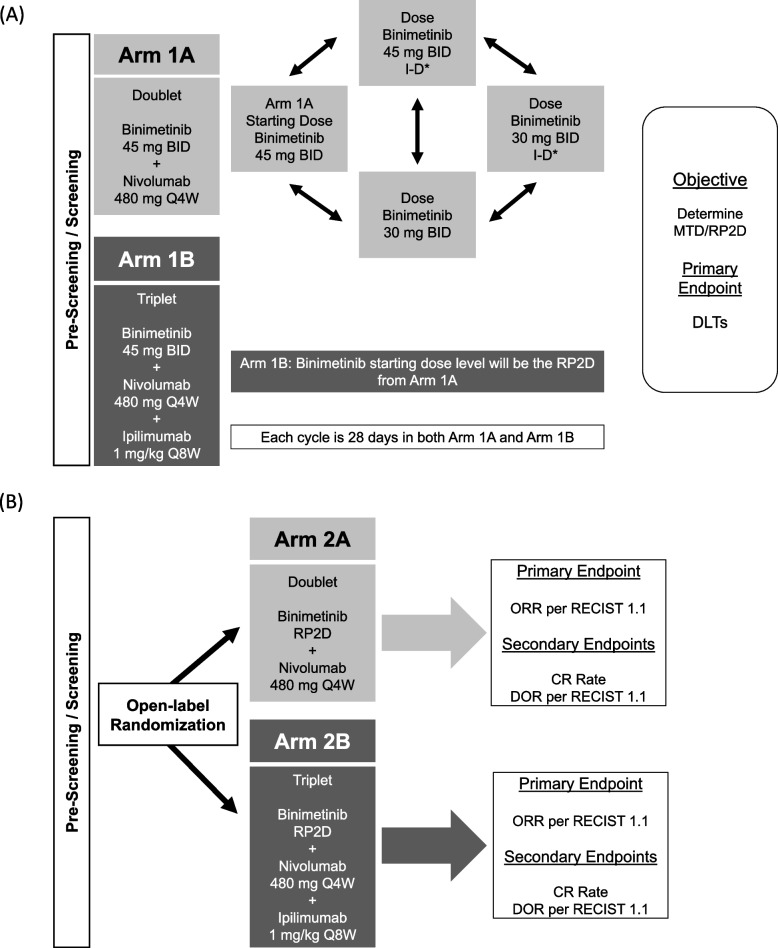
Study designs and flowcharts of **(A)** phase 1b, to determine the MTD and RP2D of binimetinib in combination with nivolumab (Arm 1A [Doublet]) and binimetinib in combination with nivolumab and ipilimumab (Arm 1B [Triplet]) and **(B)** phase 2 to determine the safety and clinical activity of the RP2D of binimetinib in combination with nivolumab (Arm 2A [Doublet]) and binimetinib in combination with nivolumab and ipilimumab (Arm 2B [Triplet]). BID, twice daily; DLT, dose-limiting toxicity; I-D, intermittent dosing; RP2D, recommended phase 2 dose; Q4W, every 4 weeks; Q8W, every 8 weeks *Three weeks on, one week off

ARRAY-162–202 (NCT03271047) is a multicenter, open-label, phase 1b/2 study to evaluate the safety and preliminary antitumor activity of binimetinib in combination with nivolumab or nivolumab and ipilimumab in adult participants with MSS mCRC and a documented *RAS* mutation who have received 1 or 2 prior lines of therapy. This study included a dose-finding phase 1b period to determine the MTD and RP2D of binimetinib, followed by a randomized phase 2 period to assess the antitumor activity of the combinations **(**Fig. 1**)**. Both phases also assessed the safety, efficacy, and pharmacokinetics of binimetinib administered in combination with nivolumab or nivolumab and ipilimumab. Key eligibility criteria included participants who: (1) were ≥ 18 years of age, (2) had Eastern Cooperative Oncology Group performance status of 0 or 1, (3) had mCRC categorized as MSS by immunohistochemistry or polymerase chain reaction-based local assay at any time prior to screening or by the central laboratory, (4) had *RAS* mutation assessed per local assay at any time prior to screening or by the central laboratory, and (5) had received ≤ 2 prior lines of systemic therapy in the metastatic setting (maintenance therapy given in the metastatic setting was not considered a separate regimen). Key exclusion criteria included: (1) prior treatment with any MEK inhibitor; (2) prior treatment with an anti–PD-1, anti–PD-L1, anti–PD-L2, anti-CD137, or anti-CTLA-4 antibody, or any other antibody or drug specifically targeting T-cell costimulation or checkpoint pathways; (3) any untreated central nervous system (CNS) lesions, unless all known CNS lesions had been treated with radiotherapy or surgery and participants remained without evidence of CNS disease progression ≥ 4 weeks after treatment, and participants must have been off corticosteroid therapy for ≥ 3 weeks; and (4) participants who had an active, known or suspected autoimmune disease.

### Study objectives and endpoints

The objective of the phase 1b part was to determine the MTD and RP2D of binimetinib administered in combination with nivolumab or nivolumab and ipilimumab. The MTD was defined as the highest combination drug dosage not causing medically unacceptable dose-limiting toxicities (DLTs) in > 35% of treated participants in the first cycle of treatment, based on a modified toxicity probability interval (mTPI-2) design. Four dose levels were tested using the mTPI-2 design, a model-based approach that is guided by a prespecified decision matrix that recommends escalating, reducing, or maintaining the same dose, or stopping dose escalation based on the number of patients with DLTs observed in the dose level under evaluation. DLTs were defined as adverse events (AEs) or clinically significant abnormal laboratory values assessed as unrelated to disease, disease progression, intercurrent illness, or concomitant medications that occurred within the first 28 days of treatment and resulted in the inability to tolerate 75% dose intensity ([administered dose in mg/planned dose in mg] × 100) of binimetinib. The primary endpoint of the phase 1b part was the incidence of DLTs resulting from binimetinib in combination with nivolumab or nivolumab and ipilimumab.

The objective of the phase 2 part was to assess the preliminary antitumor activity of the treatment combinations based on Response Evaluation Criteria in Solid Tumors (RECIST) version 1.1 with a primary endpoint of overall response rate (ORR) per RECIST 1.1. The secondary safety endpoints for both parts were the incidence and severity of AEs graded according to the National Cancer Institute Common Terminology Criteria for Adverse Events, version 4.03. The phase 1b secondary efficacy endpoint was ORR per RECIST 1.1. The phase 1b and phase 2 secondary efficacy endpoints were duration of response (DOR) and rate of complete response per RECIST 1.1.

### Study treatment and procedures

In phase 1b, Arm 1A participants received a starting dose of binimetinib 45 mg BID plus nivolumab 480 mg once every 4 weeks (Q4W), the US Food and Drug Administration–approved doses of both agents at the time (Fig. 1A). In phase 1b, Arm 1B participants received a starting dose of binimetinib 45 mg BID plus nivolumab 480 mg Q4W and ipilimumab 1 mg/kg once every 8 weeks (Q8W) (Fig. 1A). In Arm 1A and Arm 1B, dose de-escalation was planned as needed until the MTD/RP2D was determined.

In phase 2, Arm 2A participants received the MTD/RP2D of binimetinib, as determined during phase 1b, plus nivolumab 480 mg Q4W (Fig. 1B). In phase 2, Arm 2B participants received the MTD/RP2D of binimetinib, as determined during phase 1b, plus nivolumab 480 mg Q4W and ipilimumab 1 mg/kg Q8W (Fig. 1B).

Tumor response was evaluated locally by the investigator according to RECIST 1.1, using computed tomography scans and/or magnetic resonance imaging with intravenous contrast. Scans were performed at screening/baseline and then every 8 weeks (two 28-day cycles) until disease progression.

Safety was assessed throughout the study, and AEs were coded using Medical Dictionary for Regulatory Activities, version 21.0, terminology. Toxicity was assessed according to National Cancer Institute Common Terminology Criteria for Adverse Events 4.03. Participants continued treatment until disease progression, development of unacceptable toxicity, or withdrawal of informed consent.

### Statistical analysis

The MTD/RP2D of the combination treatment was estimated based on the anticipated probability of DLTs in cycle 1 for participants in the dose-determining set, which consisted of all phase 1b participants who met specified minimum exposure criteria and had sufficient safety evaluations during cycle 1 or who discontinued earlier due to a DLT during cycle 1. Efficacy analyses included all participants who received at least one dose of study drug. The ORR was calculated within each treatment arm and with exact (Clopper-Pearson) 2-sided 90% and 95% confidence intervals (CIs). A similar analysis was provided for the rate of complete response. An estimate of the DOR was presented descriptively. For progression-free survival (PFS) and OS, the survival function was constructed using the Kaplan–Meier (product-limit) method. The 25%, median, and 75% PFS and OS (in months) were summarized along with 95% CIs. Kaplan–Meier estimates with 95% CIs at specific time points were summarized as well. The safety set, which consists of all participants who received ≥ 1 dose of any study drug, was used for summaries of safety data, except for DLTs for which the dose-determining set was used. Descriptive statistics were used to summarize safety data. Statistical analyses were done using SAS, version 9.4.

## Results

A total of 75 participants were enrolled and dosed in the study: 10 participants in Arm 1A (starting dose of binimetinib 45 mg BID plus nivolumab 480 mg Q4W), 11 participants in Arm 1B (starting dose of binimetinib 45 mg BID plus nivolumab 480 mg Q4W and ipilimumab 1 mg/kg Q8W), and 27 participants each in Arm 2A (MTD/RP2D of binimetinib plus nivolumab 480 mg Q4W) and Arm 2B (MTD/RP2D of binimetinib plus nivolumab 480 mg Q4W and ipilimumab 1 mg/kg Q8W) (Table 1). In the Doublet Arms (Arms 1A and 2A) the median age at enrollment was 60 years (range, 31–80 years) (Table 1). In the Triplet Arms (Arms 1B and 2B) the median age at enrollment was 59.5 years (range, 29 − 78 years) (Table 1). Participant demographics and baseline disease characteristics were generally similar across treatment groups in the phase 1b and phase 2 parts (Table 1).

**Table 1:** Participant demographics and baseline characteristics

	**Doublet Arms**			**Triplet Arms**		
	**Arm 1A (*n* = 10)**	**Arm 2A (*n* = 27)**	**Pooled (*n* = 37)**	**Arm 1B (*n* = 11)**	**Arm 2B (*n* = 27)**	**Pooled (*n* = 38)**
Age at screening, years						
Mean (SD)	62.6 (9.17)	57.8 (11.80)	59.1 (11.24)	56.5 (10.02)	57.8 (12.07)	57.4 (11.39)
Median (min, max)	63.5 (44, 74)	59.0 (31, 80)	60.0 (31, 80)	59.0 (41, 71)	61.0 (29, 78)	59.5 (29, 78)
Age group at screening, n (%)						
< 65 years	5 (50.0)	19 (70.4)	24 (64.9)	9 (81.8)	20 (74.1)	29 (76.3)
≥ 65 years	5 (50.0)	8 (29.6)	13 (35.1)	2 (18.2)	7 (25.9)	9 (23.7)
Sex, n (%)						
Male	8 (80.0)	16 (59.3)	24 (64.9)	7 (63.6)	17 (63.0)	24 (63.2)
Female	2 (20.0)	11 (40.7)	13 (35.1)	4 (36.4)	10 (37.0)	14 (36.8)
Race, n (%)						
White	9 (90.0)	21 (77.8)	30 (81.1)	11 (100)	23 (85.2)	34 (89.5)
Black or African American	1 (10.0)	1 (3.7)	2 (5.4)	0	1 (3.7)	1 (2.6)
Asian	0	1 (3.7)	1 (2.7)	0	0	0
Other	0	4 (14.8)	4 (10.8)	0	3 (11.1)	3 (7.9)
Ethnicity, n (%)						
Hispanic/Latino	0	1 (3.7)	1 (2.7)	0	0	0
Not Hispanic/Latino	10 (100)	21 (77.8)	31 (83.8)	11 (100)	23 (85.2)	34 (89.5)
Unknown	0	1 (3.7)	1 (2.7)	0	0	0
Not reported	0	4 (14.8)	4 (10.8)	0	4 (14.8)	4 (10.5)
ECOG PS n (%)						
0	7 (70.0)	13 (48.1)	20 (54.1)	3 (27.3)	11 (40.7)	14 (36.8)
1	3 (30.0)	14 (51.9)	17 (45.9)	8 (72.7)	16 (59.3)	24 (63.2)

*ECOG PS* Eastern Cooperative Oncology Group performance status, *max* maximum, *min* minimum

### MTD/RP2D

In Arm 1A (Doublet), of the 9 participants (90.0%) evaluable for DLTs, 1 participant (11.1%) was reported to have grade 3 dermatitis acneiform. In Arm 1B (Triplet), 2 of 11 participants (18.2%) reported multiple DLTs. One participant had DLTs of grade 3 rash, grade 2 blurred vision, and grade 2 pneumonitis, all reported during the first treatment cycle. Another participant had a DLT of grade 3 colitis reported during the first treatment cycle. The MTD and RP2D of binimetinib were determined as 45 mg BID for both Arm 1A (Doublet) and Arm 2A (Triplet). Therefore, this dose was used in the phase 2 part of the study (Arm 1B and Arm 2B).

### Safety

In the Doublet Arms, binimetinib and nivolumab exposure had a median duration of 3.1 months (range, 1.8–7.1 months) and 3.2 months (range, 1.8–7.6 months) in Arm 1A and 2.0 months (range, 0.03–23.8 months) and 2.0 months (range, 0.0–24.5 months) in Arm 2A. In the Triplet Arms, binimetinib and nivolumab exposure had a median duration of 2.8 months (range, 0.7–5.6 months) and 2.8 months (range, 0.9–6.3 months) in Arm 1B and 2.1 months (range, 0.4–17.5 months) and 2.8 months (range, 0.9–17.9) in Arm 2B. The median duration of ipilimumab exposure was 3.7 months (range, 1.8–6.2 months) in Arm 1B and 1.8 months (range, 1.8–18.9 months) in Arm 2B. The most frequently reported AEs are described in Table S1.

In the Doublet Arms, the most frequently reported treatment-related AEs were dermatitis acneiform (51.4%), blood creatine phosphokinase increased (48.6%), diarrhea (45.9%), fatigue (29.7%), and edema peripheral (29.7%) (Table 2). The most frequently reported treatment-related AEs in the Triplet Arms were dermatitis acneiform (47.4%), blood creatine phosphokinase increased (39.5%), diarrhea (39.5%), rash (39.5%), fatigue (28.9%), nausea (28.9%), and vomiting (28.9%) (Table 2).

**Table 2:** Treatment-related adverse events experienced by ≥ 15% of participants in ≥ 1 arm by preferred term (safety set, phase 1b/2)

	**Doublet Arms (Arms 1A and 2A) *n* = 37**	**Triplet Arms (Arms 1B and 2B) *n* = 38**
**TRAEs, n (%)**		
Dermatitis acneiform	19 (51.4)	18 (47.4)
Blood creatine phosphokinase increased	18 (48.6)	15 (39.5)
Diarrhea	17 (45.9)	15 (39.5)
Rash	8 (21.6)	15 (39.5)
Fatigue	11 (29.7)	11 (28.9)
Nausea	10 (27.0)	11 (28.9)
Edema peripheral	11 (29.7)	9 (23.7)
Vomiting	8 (21.6)	11 (28.9)
Decreased appetite	10 (27.0)	8 (21.1)
Asthenia	8 (21.6)	7 (18.4)
Pruritus	5 (13.5)	10 (26.3)
Ejection fraction decreased	5 (13.5)	7 (18.4)
Aspartate aminotransferase increased	3 (8.1)	7 (18.4)
Dry skin	2 (5.4)	7 (18.4)
Pyrexia	2 (5.4)	7 (18.4)
Alanine aminotransferase increased	2 (5.4)	6 (15.8)
Cough	1 (2.7)	6 (15.8)
Dry mouth	2 (5.4)	5 (13.2)
Dyspnea	3 (8.1)	4 (10.5)
Dysgeusia	1 (2.7)	4 (10.5)
Visual impairment	2 (5.4)	3 (7.9)
Dizziness	2 (5.4)	2 (5.3)
Pneumonitis	0	4 (10.5)
Rash pruritic	3 (8.1)	0

*TRAE* Treatment-related adverse event

AEs related to binimetinib were observed including grade 3 exfoliative rash, grade 3 alanine aminotransferase increased, grade 2 cardiac failure congestive, and grade 1 ejection fraction decreased that were reported for one participant each. One participant reported AEs related to binimetinib, nivolumab, and ipilimumab of grade 2 maculopathy and grade 2 retinopathy. Serious AEs related to all 3 study drugs were also reported. One participant had a grade 3 skin reaction, grade 3 myocarditis, grade 2 pleurisy, and grade 2 pneumonitis related to nivolumab and ipilimumab. Another participant had grade 3 colitis and 1 participant had grade 3 pancreatitis and grade 3 transaminase increased related to binimetinib, nivolumab, and ipilimumab. Additionally, 1 serious AE related to binimetinib, nivolumab, and ipilimumab of grade 5 *Pneumocystis jirovecii* pneumonia occurred in Arm 1B (Triplet) and 1 serious AE of grade 5 empyema occurred in Arm 2B (Triplet).

All-causality immune-mediated AEs were reported for 22 participants (59.5%) in the Doublet Arms and 25 participants (65.8%) in the Triplet Arms. The most frequently (≥ 4 participants) reported all-causality immune-mediated AEs were diarrhea, fatigue, and dermatitis acneiform in the Doublet Arms, and diarrhea, fatigue, dermatitis acneiform, rash, and pruritus in the Triplet Arms.

A total of 16 participants (21.3%) reported AEs leading to discontinuation of any study drug. All-causality AEs leading to discontinuation of binimetinib were reported for 3 participants (8.1%) in the Doublet Arms and 13 participants (34.2%) in Triplet Arms. Two participants in the Doublet Arms permanently discontinued binimetinib due to the following all-causality AEs (reported for 1 participant each): an AE of grade 4 blood bilirubin increased that was not considered treatment related and a serious AE of grade 4 myocarditis that was considered related to nivolumab. There was also a serious AE of grade 5 acute coronary syndrome that was not considered treatment related. Thirteen participants (34.2%) in the Triplet Arms permanently discontinued binimetinib due to the following all-causality AEs (reported for 1 participant each): grade 4 bacterial sepsis, grade 4 pneumonia, and grade 3 abdominal infection. None of these events were considered treatment related.

### Clinical activity

In the phase 1b part, no participants had a response to binimetinib in combination with nivolumab in Arm 1A (Doublet) or binimetinib in combination with nivolumab and ipilimumab in Arm 1B (Triplet). In Arm 1A, 6 participants (60%) had stable disease (SD), for a disease control rate (DCR) of 60% (95% CI: 26.2, 87.8), and in Arm 1B, 4 participants (36.4%) had SD, for a DCR of 36.4% (95% CI: 10.9, 69.2) (Table 3). In the phase 2 part (Arm 2A [Doublet] and Arm 2B [Triplet]), the confirmed ORR was 0% in Arm 2A and 7.4% (90% CI: 1.3, 21.5) in Arm 2B, with 2 participants having a confirmed partial response (Table 3). Furthermore, 11 participants (40.7%) in Arm 2A had SD, for a DCR of 40.7% (95% CI: 22.4, 61.2); 13 participants (48.1%) in Arm 2B had SD, for a DCR of 55.6% (95% CI: 35.3, 74.5) (Table 3). For the 2 participants in Arm 2B who had a partial response (Table 3) the DORs were 462 and 229 days.

**Table 3:** Summary of best overall response per RECIST 1.1 criteria (full analysis set, phase 1b/2)

	**Doublet Arms**			**Triplet Arms**		
	**Arm 1A** (**n = 10)**	**Arm 2A** (**n = 27)**	**Pooled** **(n = 37)**	**Arm 1B** (**n = 11)**	**Arm (2B** **n = 27)**	**Pooled (n = 38)**
Best overall response, n (%)^a, b^						
CR	0	0	0	0	0	0
PR	0	0	0	0	2 (7.4)	2 (5.3)
SD	6 (60.0)	11 (40.7)	17 (45.9)	4 (36.4)	13 (48.1)	17 (44.7)
PD	4 (40.0)	15 (55.6)	19 (51.4)	3 (27.3)	10 (37.0)	13 (34.2)
Not evaluable	0	1 (3.7)	1 (2.7)	4 (36.4)	2 (7.4)	6 (15.8)
Overall response rate (CR + PR), n (%)^b^ [90% CI] [95% CI]	0	0	0	0	2 (7.4) [1.3, 21.5] [0.9, 24.3]	2 (5.3) [0.9, 15.7] [0.6, 17.7]
Disease control rate (CR + PR + SD), n (%) [95% CI]	6 (60.0) [26.2, 87.8]	11 (40.7) [22.4, 61.2]	17 (45.9) [29.5, 63.1]	4 (36.4) [10.9, 69.2]	15 (55.6) [35.3, 74.5]	19 (50.0) [33.4, 66.6]

*CR* Complete response, *PD* Progressive disease, *PR* Partial response, *SD* Stable disease

^a^Best overall response is based on investigator's assessment using Response Evaluation Criteria in Solid Tumors version 1.1.

^b^Confirmed

In the Doublet Arms, the median PFS, defined as the time from start of treatment to the date of the first documented disease progression or death due to any cause, was 3.0 months (95% CI: 1.6, 3.7) in Arm 1A (*n *= 10) and 1.8 months (95% CI: 1.7, 3.7) in Arm 2A (*n* = 27). In the Triplet Arms, the median PFS was 2.4 months (95% CI: 1.7, 5.6) in Arm 1B (*n* = 11) and 3.0 months (95% CI: 1.8, 13.8) in Arm 2B (*n* = 27). The median OS, defined as the duration from the start of treatment to the time of death due to any cause, was 5.1 months (95% CI: 2.5, 22.0) in Arm 1A, 7.6 months (95% CI: 4.1, 10.6) in Arm 2A, 5.1 months (95% CI: 1.7, 16.7) in Arm 1B, and 12.0 months (95% CI: 8.3, 17.8) in Arm 2B.

## Discussion

Aside from chemotherapy, which has limited efficacy, treatment options are lacking for previously treated patients with *RAS*-mutated MSS mCRC. Therefore, we undertook this phase 1b/2 study to determine if the combination of binimetinib with nivolumab or nivolumab and ipilimumab could improve outcomes for these patients, as the combination of MEK inhibition with ICI therapy has shown evidence of tumor regression even where either agent alone was only modestly effective [14, 23–25].

In this study, the safety profile of the RP2D triplet regimen of binimetinib, nivolumab, and ipilimumab was similar to the clinically accepted and approved doublet regimen of nivolumab and ipilimumab [20, 26, 27], indicating that these therapies can be given together [16, 25, 28]. Moreover, the observed AEs for binimetinib were consistent with those reported for other MEK1/2 inhibitors, which were reversible with appropriate supportive medical care or dose modifications [16, 25, 28]. However, based on the ORR results, adding binimetinib in combination with nivolumab in Arm 1A or Arm 2A (Doublet Arms) did not result in an additional clinical benefit. This was also the case when binimetinib was added to nivolumab and ipilimumab in Arm 1B or Arm 2B (Triplet Arms).

Even though this study was able to examine several combinations at once, data interpretation was limited due to the small number of participants in each arm of the phase 1B and phase 2 parts. Furthermore, while no tissue samples were collected to look at possible modes of action, it could be that the extent and duration of MAPK pathway inhibition was insufficient to alter the immune environment or that the preclinical data on which this study design was based was not as relevant in the real world in this patient population. Prior studies have highlighted the challenges associated with treating patients with MSS mCRC with ICI therapy [28–31]. The combination of MEK inhibition and ICI therapy might not be sufficient to overcome the “immune cold” nature of the tumor microenvironment associated with MSS mCRC [32, 33]. In addition, alternative mechanisms that bypass the inhibition of the MAPK pathway by a MEK inhibitor in MSS mCRC could contribute to the lack of additional clinical benefit from adding binimetinib to ICI therapy [34]. Despite the lack of efficacy results in this study, MEK inhibitors still have promise in MSS mCRC and are currently being tested with broader inhibitors upstream of MEK1/2 in the RAS-regulated RAF–MEK1/2–ERK pathway, including drugs targeting SHP2 and SOS [35, 36]. MEK inhibitors are also being tested with cell cycle inhibitors, such as drugs targeting CDK4/6, to determine their safety and effectiveness [37].

## Conclusions

As there are currently approved therapies available for patients with previously treated MSS mCRC with *RAS* mutation that have demonstrated a survival benefit [38, 39], the lack of clinical benefit with the doublet and triplet regimens in the current study does not justify further clinical development in patients with CRC. However, other ongoing MEK inhibitor combination studies may have promise for patients with *RAS*-mutated MSS CRC.

### Supplementary Information

**Supplementary Material 1. **

## Data Availability

Upon request, and subject to review, Pfizer will provide the data that support the findings of this study. Subject to certain criteria, conditions, and exceptions, Pfizer may also provide access to the related individual de-identified participant data. See https://www.pfizer.com/science/clinical-trials/trial-data-and-results for more information.

## Abbreviations

AE: Adverse event
BID: Twice daily
CNS: Central nervous system
CRC: Colorectal cancer
DCR: Disease control rate
DLT: Dose-limiting toxicity
dMMR: Mismatch repair deficient
DOR: Duration of response
ICI: Immune checkpoint inhibitor
mAb: Monoclonal antibody
mCRC: Metastatic colorectal cancer
MSI-H: Microsatellite instability high
MSS: Microsatellite stable
MTD: Maximum tolerable dose
mTPI: Modified toxicity probability interval
ORR: Overall response rate
OS: Overall survival
PFS: Progression-free survival
Q4W: Every 4 weeks
Q8W: Every 8 weeks
RECIST: Response Evaluation Criteria in Solid Tumors
RP2D: Recommended phase 2 dose
SD: Stable disease

## References

[1] Sung H, Ferlay J, Siegel RL, Laversanne M, Soerjomataram I, Jemal A (2021). Global Cancer Statistics 2020: GLOBOCAN estimates of incidence and mortality worldwide for 36 cancers in 185 countries. CA Cancer J Clin.

[2] Siegel RL, Miller KD, Goding Sauer A, Fedewa SA, Butterly LF, Anderson JC (2020). Colorectal cancer statistics, 2020. CA Cancer J Clin.

[3] Chakrabarti S, Peterson CY, Sriram D, Mahipal A (2020). Early stage colon cancer: current treatment standards, evolving paradigms, and future directions. World J Gastrointest Oncol.

[4] Singh MP, Rai S, Pandey A, Singh NK, Srivastava S (2021). Molecular subtypes of colorectal cancer: an emerging therapeutic opportunity for personalized medicine. Genes Dis.

[5] Cancer Genome Access Network (2012). Comprehensive molecular characterization of human colon and rectal cancer. Nature.

[6] Zhang B, Wang J, Wang X, Zhu J, Liu Q, Shi Z (2014). Proteogenomic characterization of human colon and rectal cancer. Nature.

[7] Serebriiskii IG, Connelly C, Frampton G, Newberg J, Cooke M, Miller V (2019). Comprehensive characterization of RAS mutations in colon and rectal cancers in old and young patients. Nat Commun.

[8] Le DT, Uram JN, Wang H, Bartlett BR, Kemberling H, Eyring AD (2015). PD-1 blockade in tumors with mismatch-repair deficiency. N Engl J Med.

[9] Kim CW, Chon HJ, Kim C (2021). Combination immunotherapies to overcome intrinsic resistance to checkpoint blockade in microsatellite stable colorectal cancer. Cancers (Basel).

[10] Giordano G, Parcesepe P, D'Andrea MR, Coppola L, Di Raimo T, Remo A (2019). JAK/Stat5-mediated subtype-specific lymphocyte antigen 6 complex, locus G6D (LY6G6D) expression drives mismatch repair proficient colorectal cancer. J Exp Clin Cancer Res.

[11] Benvenuti S, Sartore-Bianchi A, Di Nicolantonio F, Zanon C, Moroni M, Veronese S (2007). Oncogenic activation of the RAS/RAF signaling pathway impairs the response of metastatic colorectal cancers to anti-epidermal growth factor receptor antibody therapies. Cancer Res.

[12] Taieb J, Le Malicot K, Shi Q, Penault-Llorca F, Bouche O, Tabernero J (2016). Prognostic value of BRAF and KRAS mutations in MSI and MSS stage III colon cancer. J Natl Cancer Inst..

[13] Dienstmann R, Connor K, Byrne AT, COLOSSUS Consortium (2020). Precision therapy in RAS mutant colorectal cancer. Gastroenterology..

[14] Ebert PJR, Cheung J, Yang Y, McNamara E, Hong R, Moskalenko M (2016). MAP kinase inhibition promotes T cell and anti-tumor activity in combination with PD-L1 checkpoint blockade. Immunity.

[15] Liu L, Mayes PA, Eastman S, Shi H, Yadavilli S, Zhang T (2015). The BRAF and MEK inhibitors dabrafenib and trametinib: effects on immune function and in combination with immunomodulatory antibodies targeting PD-1, PD-L1, and CTLA-4. Clin Cancer Res.

[16] Bendell JC, Javle M, Bekaii-Saab TS, Finn RS, Wainberg ZA, Laheru DA (2017). A phase 1 dose-escalation and expansion study of binimetinib (MEK162), a potent and selective oral MEK1/2 inhibitor. Br J Cancer.

[17] Kopetz S, Grothey A, Yaeger R, Van Cutsem E, Desai J, Yoshino T (2019). Encorafenib, binimetinib, and cetuximab in BRAF V600E-mutated colorectal cancer. N Engl J Med.

[18] Friedrich T, Blatchford PJ, Lentz RW, Davis SL, Kim SS, Leal AD (2022). A phase II study of pembrolizumab, binimetinib, and bevacizumab in patients with microsatellite-stable, refractory, metastatic colorectal cancer (mCRC). J Clin Oncol..

[19] Cherri S, Oneda E, Zanotti L, Zaniboni A (2023). Optimizing the first-line treatment for metastatic colorectal cancer. Front Oncol.

[20] Overman MJ, Lonardi S, Wong KYM, Lenz HJ, Gelsomino F, Aglietta M (2018). Durable clinical benefit with nivolumab plus ipilimumab in DNA mismatch repair-deficient/microsatellite instability-high metastatic colorectal cancer. J Clin Oncol.

[21] Overman MJ, McDermott R, Leach JL, Lonardi S, Lenz HJ, Morse MA (2017). Nivolumab in patients with metastatic DNA mismatch repair-deficient or microsatellite instability-high colorectal cancer (CheckMate 142): an open-label, multicentre, phase 2 study. Lancet Oncol.

[22] Chirica M, Le Bourhis L, Lehmann-Che J, Chardiny V, Bouhidel F, Foulboeuf L (2015). Phenotypic analysis of T cells infiltrating colon cancers: correlations with oncogenetic status. Oncoimmunology.

[23] Mimura K, Kua LF, Shiraishi K, Kee Siang L, Shabbir A, Komachi M (2014). Inhibition of mitogen-activated protein kinase pathway can induce upregulation of human leukocyte antigen class I without PD-L1-upregulation in contrast to interferon-gamma treatment. Cancer Sci.

[24] Le DT, Kim TW, Van Cutsem E, Geva R, Jager D, Hara H (2020). Phase II open-label study of pembrolizumab in treatment-refractory, microsatellite instability-high/mismatch repair-deficient metastatic colorectal cancer: KEYNOTE-164. J Clin Oncol.

[25] Dummer R, Lebbé C, Atkinson V, Mandala M, Nathan PD, Arance A (2020). Combined PD-1, BRAF and MEK inhibition in advanced BRAF-mutant melanoma: safety run-in and biomarker cohorts of COMBI-i. Nat Med.

[26] Larkin J, Chiarion-Sileni V, Gonzalez R, Grob JJ, Cowey CL, Lao CD (2015). Combined nivolumab and ipilimumab or monotherapy in untreated melanoma. N Engl J Med.

[27] Wolchok JD, Kluger H, Callahan MK, Postow MA, Rizvi NA, Lesokhin AM (2013). Nivolumab plus ipilimumab in advanced melanoma. N Engl J Med.

[28] Hellmann MD, Kim TW, Lee CB, Goh BC, Miller WH, Oh DY (2019). Phase Ib study of atezolizumab combined with cobimetinib in patients with solid tumors. Ann Oncol.

[29] Eng C, Kim TW, Bendell J, Argiles G, Tebbutt NC, Di Bartolomeo M (2019). Atezolizumab with or without cobimetinib versus regorafenib in previously treated metastatic colorectal cancer (IMblaze370): a multicentre, open-label, phase 3, randomised, controlled trial. Lancet Oncol.

[30] Lin KX, Istl AC, Quan D, Skaro A, Tang E, Zheng X (2023). PD-1 and PD-L1 inhibitors in cold colorectal cancer: challenges and strategies. Cancer Immunol Immunother.

[31] Johnson B, Haymaker CL, Parra ER, Soto LMS, Wang X, Thomas JV (2022). Phase II study of durvalumab (anti-PD-L1) and trametinib (MEKi) in microsatellite stable (MSS) metastatic colorectal cancer (mCRC). J Immunother Cancer.

[32] Ros J, Balconi F, Baraibar I, Saoudi Gonzalez N, Salva F, Tabernero J (2023). Advances in immune checkpoint inhibitor combination strategies for microsatellite stable colorectal cancer. Front Oncol.

[33] Sahin IH, Ciombor KK, Diaz LA, Yu J, Kim R (2022). Immunotherapy for microsatellite stable colorectal cancers: challenges and novel therapeutic avenues. Am Soc Clin Oncol Educ Book.

[34] Weng J, Li S, Zhu Z, Liu Q, Zhang R, Yang Y, Li X (2022). Exploring immunotherapy in colorectal cancer. J Hematol Oncol.

[35] Fedele C, Ran H, Diskin B, Wei W, Jen J, Geer MJ (2018). SHP2 inhibition prevents adaptive resistance to MEK inhibitors in multiple cancer models. Cancer Discov.

[36] Sheffels E, Kortum RL (2021). Breaking oncogene addiction: getting RTK/RAS-mutated cancers off the SOS. J Med Chem.

[37] Sorokin AV, Kanikarla Marie P, Bitner L, Syed M, Woods M, Manyam G (2022). Targeting RAS mutant colorectal cancer with dual inhibition of MEK and CDK4/6. Cancer Res.

[38] Grothey A, Van Cutsem E, Sobrero A, Siena S, Falcone A, Ychou M (2013). Regorafenib monotherapy for previously treated metastatic colorectal cancer (CORRECT): an international, multicentre, randomised, placebo-controlled, phase 3 trial. Lancet.

[39] Prager GW, Taieb J, Fakih M, Ciardiello F, Van Cutsem E, Elez E (2023). Trifluridine-tipiracil and bevacizumab in refractory metastatic colorectal cancer. N Engl J Med.

