# Supplementary material for: Binimetinib in combination with nivolumab or nivolumab and ipilimumab in patients with previously treated microsatellite-stable metastatic colorectal cancer with RAS mutations in an open-label phase 1b/2 study
Source: BMC Cancer. 2024 Apr 11;24:446. doi: 10.1186/s12885-024-12153-5 (PMC11007903; doi:10.1186/s12885-024-12153-5)
Supplement: Supplementary file 1 — Supplementary Material 1. [file 12885_2024_12153_MOESM1_ESM.docx]

**Table S1.** Adverse events, regardless of study drug relationship, and select laboratory abnormalities occurring in ≥20% of participants across treatment groups.

|  | **Doublet Arms**  **(Arms 1A and 2A)**  **n=37** | **Triplet Arms**  **(Arms 1B and 2B)**  **n=38** |
| --- | --- | --- |
| **AEs, n (%)** |  |  |
| Diarrhea | 20 (54.1) | 19 (50.0) |
| Dermatitis acneiform | 20 (54.1) | 18 (47.4) |
| Blood creatine phosphokinase increased | 20 (54.1) | 16 (42.1) |
| Fatigue | 15 (40.5) | 17 (44.7) |
| Nausea | 17 (45.9) | 13 (34.2) |
| Edema peripheral | 13 (35.1) | 16 (42.1) |
| Pyrexia | 11 (29.7) | 17 (44.7) |
| Vomiting | 9 (24.3) | 18 (47.4) |
| Rash | 11 (29.7) | 15 (39.5) |
| Decreased appetite | 15 (40.5) | 9 (23.7) |
| Asthenia | 10 (27.0) | 10 (26.3) |
| Constipation | 11 (29.7) | 9 (23.7) |
| Cough | 8 (21.6) | 9 (23.7) |
| Aspartate aminotransferase increased | 3 (8.1) | 12 (31.6) |
| Pruritus | 5 (13.5) | 10 (26.3) |
| Abdominal pain | 6 (16.2) | 6 (15.8) |
| Alanine aminotransferase  increased | 2 (5.4) | 10 (26.3) |
| Anemia | 7 (18.9) | 5 (13.2) |
| Dyspnea | 4 (10.8) | 8 (21.1) |
| Ejection fraction decreased | 5 (13.5) | 7 (18.4) |
| Dry skin | 3 (8.1) | 7 (18.4) |
| Back pain | 5 (13.5) | 4 (10.5) |
| Dry mouth | 3 (8.1) | 6 (15.8) |
| Stomatitis | 4 (10.8) | 5 (13.2) |
| Hypokalemia | 5 (13.5) | 3 (7.9) |
| Blood alkaline phosphatase increased | 2 (5.4) | 5 (13.2) |
| Dysgeusia | 2 (5.4) | 4 (10.5) |
| Gastroesophageal reflux disease | 1 (2.7) | 5 (13.2) |
| Myalgia | 3 (8.1) | 3 (7.9) |
| Visual impairment | 2 (5.4) | 4 (10.5) |
| Pneumonitis | 1 (2.7) | 4 (10.5) |
| Dizziness | 2 (5.4) | 2 (5.3) |
| Influenza like illness | 1 (2.7) | 2 (5.3) |
| Rales | 1 (2.7) | 2 (5.3) |
| Rash pruritic | 3 (8.1) | 0 |
| Confusional state | 0 | 2 (5.3) |

AE, adverse event.
